# Preclinical Considerations for Long-acting Delivery of Tenofovir Alafenamide from Subdermal Implants for HIV Pre-exposure Prophylaxis

**DOI:** 10.1007/s11095-022-03440-6

**Published:** 2022-11-23

**Authors:** Manjula Gunawardana, Mariana Remedios-Chan, Debbie Sanchez, Rob Fanter, Simon Webster, Paul Webster, John A. Moss, MyMy Trinh, Martin Beliveau, Christina M. Ramirez, Mark A. Marzinke, Joseph Kuo, Philippe A. Gallay, Marc M. Baum

**Affiliations:** 1 Department of Chemistry, Oak Crest Institute of Science, 128-132 W. Chestnut Ave., Monrovia, CA USA; 2 Certara USA, Inc., Integrated Drug Development, 100 Overlook Center, Suite 101, Princeton, NJ USA; 3 Department of Biostatistics, UCLA Fielding School of Public Health, University of California, Los Angeles (UCLA), 650 Charles E. Young Drive, Los Angeles, CA USA; 4 Department of Medicine, Johns Hopkins University, 600 N. Wolfe Street, Baltimore, MD USA; 5 Department of Pathology, Johns Hopkins University, 600 N. Wolfe Street/Carnegie 417, Baltimore, MD USA; 6 Department of Immunology & Microbiology, The Scripps Research Institute, 10550 North Torrey Pines Road, La Jolla, CA USA

**Keywords:** comparative animal models, HIV pre-exposure prophylaxis, reservoir subdermal implant, tenofovir alafenamide

## Abstract

**Purpose:**

Long-acting formulations of the potent antiretroviral prodrug tenofovir alafenamide (TAF) hold potential as biomedical HIV prevention modalities. Here, we present a rigorous comparison of three animal models, C57BL/6 J mice, beagle dogs, and merino sheep for evaluating TAF implant pharmacokinetics (PKs).

**Methods:**

Implants delivering TAF over a wide range of controlled release rates were tested *in vitro* and in mice and dogs. Our existing PK model, supported by an intravenous (IV) dosing dog study, was adapted to analyze mechanistic aspects underlying implant TAF delivery.

**Results:**

TAF *in vitro* release in the 0.13 to 9.8 mg d^−1^ range with zero order kinetics were attained. Implants with equivalent fabrication parameters released TAF in mice and sheep at rates that were not statistically different, but were 3 times higher in dogs. When two implants were placed in the same subcutaneous pocket, a two-week creep to *C*_*max*_ was observed in dogs for systemic drug and metabolite concentrations, but not in mice. Co-modeling IV and TAF implant PK data in dogs led to an apparent TAF bioavailability of 9.6 in the single implant groups (compared to the IV group), but only 1.5 when two implants were placed in the same subcutaneous pocket.

**Conclusions:**

Based on the current results, we recommend using mice and sheep, with macaques as a complementary species, for preclinical TAF implant evaluation with the caveat that our observations may be specific to the implant technology used here. Our report provides fundamental, translatable insights into multispecies TAF delivery via long-acting implants.

## Introduction

The number of annual, new HIV infections are stalling around 1.7 million in 2019 [1], and innovative biomedical solutions are needed to bridge the HIV-1 prevention gap that has been attained. To meet the ambitious Fast-Track UNAIDS strategy to end the AIDS epidemic by 2030 [2], gender-neutral therapies for sexual HIV-1 prevention in at risk populations that can simultaneously provide safe and durable protection in vaginal and rectal compartments will be required [3–7].

Long-acting drug delivery approaches such as injectable and implantable antiretroviral (ARV) formulations hold significant potential for HIV-1 pre-exposure prophylaxis (PrEP), as they overcome the adherence burden associated with frequent dosing (*e.g.*, daily oral regimens). To this end, we, and others, are developing subdermal implant technologies to deliver the potent nucleoside reverse transcriptase inhibitor prodrug tenofovir alafenamide (TAF) [8]. To date, ours is the only system to have advanced into clinical trials [9]. Implant evaluation in preclinical models forms a critical component of the drug product development phase, and holds scientific importance by investigating mechanistic questions that underpin device pharmacology. In the current report, we conduct a detailed evaluation of the pharmacokinetics (PKs) underlying TAF delivery from our implant system in dogs, and compare these findings with results obtained in mice and sheep.

## Materials and Methods

### Materials

TAF, as the free-base, was kindly provided by Gilead Sciences, Inc. (Foster City, CA). Medical-grade silicone tubing was custom-manufactured by Trelleborg Healthcare & Medical (Paso Robles, CA). All other chemicals and reagents were purchased as described previously [10], unless otherwise noted.

### Subdermal Implant Fabrication

Mouse-sized (length, 10 mm) and human-sized (length, 40 mm) TAF implants were fabricated using methods described previously [10, 11]. For preclinical studies, TAF implants were fabricated in a low bioburden environment. In some cases, they were terminally sterilized by gamma irradiation at 25 kGy (Sterigenics, Corona, CA). In others, they were cleaned with 70% v/v isopropanol using a sterile cotton swab. Implants for *in vivo* evaluation were sealed individually in moisture-barrier pouches (Technipaq, Crystal Lake, IL) for storage prior to use.

### *In Vitro* Drug Release Studies

*In vitro* release studies were designed to mimic sink conditions and were carried out in dissolution medium (100 mL), consisting of 1 × PBS with 0.01% NaN_3_, at 37°C and 72 RPM, as described previously [10].

### Residual Drug Analysis

Analysis of residual drug remaining in implants was used to measure drug purity following *in vitro* studies, and to determine TAF release rate as well as drug purity following *in vivo* studies. Residual drug analysis was performed according to published methods [10].

### Preclinical Studies

All animal studies were carried out in strict accordance with the recommendations in *the Guide for the Care and Use of Laboratory Animals of the National Institutes of Health* [12], under approved Institutional Animal Care and Use Committee (IACUC) protocols using internal Standard Operating Procedures at Sinclair Research (Auxvasse, MO) and The Scripps Research Institute (La Jolla, CA).

Beagle dog studies were conducted at Sinclair Research. In Study S14168, young adult female beagle dogs (*N* = 12), with a body weight range 8.0–13.9 kg were used. The implantation site was aseptically prepared using alternating chlorhexidine and alcohol wipes. A thin layer of topical anesthetic (2.5% lidocaine / 2.5% prilocaine cream) was applied to the clipped scapular region. Using aseptic techniques, the implant(s) was/were preloaded into a trocar prior to placement. The trocar tip was inserted into the subdermal tissue between the shoulder blades and a metal rod was used to push out the implant(s). This location was chosen for implantation in consultation with the veterinarian to minimize the risk of the dogs disturbing the site, leading to possible infection and implant removal. The skin then was pinched at the end of the trocar tip to ensure the implant(s) remained in the subdermal tissue and the tip was withdrawn from the skin. Following device implantation, the implants were gently manipulated to ensure proper placement. Four animals received a single implant, while eight animals received two implants, sequentially in the same subcutaneous pocket.

Implants were removed on Study Day 30. Using aseptic techniques to minimize contamination, the site was gently manipulated to locate the implants. Once located, an incision was made, and the implantation site exposed. Using forceps, the implant was recovered from the subdermal tissue.

Carprofen (4.4 mg kg^−1^, PO) was administered once daily on Days 0, 1, and 2 following device implantation. All animals were under general anesthesia for device removal and vaginal/rectal biopsy collections. Anesthesia was induced with propofol (6 mg kg^−1^, IV) and maintained using direct administration of isoflurane (0.5% to 5% in 100% oxygen). Prior to device removal and each biopsy collection, buprenorphine (0.005 to 0.02 mg kg^−1^, IM) was administered to each animal.

Blood was collected on Days -3, 1 (0.5, 6, and 24 h), 2, 4, 7, 10, 14, 21, 30, 31, 33, 35, and 37 for plasma and peripheral blood mononuclear cell (PBMC) isolation as described previously [10]. On Study Day 1 (1, 2, 3, and 12 h) additional blood samples were collected for plasma only. Vaginal and rectal fluid samples were collected on Study Days 1, 2, 4, 5, 10, 14, 21, 30, 31, 33, 35, and 37. Vaginal and rectal biopsies were collected on Study Days 14, 30, 33, and 37. Vaginal and rectal samples were weighted, flash-frozen in liquid nitrogen, and stored/transported at -80°C. Used TAF implant were collected and stored frozen at -80°C for analysis residual drug analysis and, hence, *in vivo* release rate calculation [10].

Animal body weights were recorded at predetermined timepoints along with clinical observations. Draize scoring of the implantation site was carried out pre-dose, once daily starting at implantation for 7 days, and weekly thereafter. Implantation site samples for microbial DNA analysis were collected at implantation and removal.

In study S15304, groups of young adult female beagle dogs (*N* = 2–3 per group) with a body weight range 8.3–10.6 kg were used. The animals either received a single TAF implant, a placebo implant, or trocar insertion and removal (no implant) and implanted devices were in place for 14 days. Procedures were similar to those described for S14168, except that no samples for bioanalysis were collected and local biopsies (*i.e.*, implant site) were collected at implant removal, and stored in neutral-buffered formalin (10% v/v) for histopathological evaluation. The study was designed to measure a range of TAF implant *in vivo* release rates, and any associated local tolerance observations, not implant PKs.

In study S14161, the product consisted of a TAF solution (0.5 mg mL^−1^) in the following vehicle: ethanol (5% v/v), polyethylene glycol (PEG) 300 (30% v/v), and water (65% v/v). The TAF solution was prepared less than 2 h before dosing. Female young adult beagle dogs (*N* = 4) with a body weight range 10.5–11.1 kg were used. A dose of 1.0 mg kg^−1^ (2 mL kg^−1^) was administered as a single IV infusion over a 30-min time period.

Blood was collected pre-dose, at 0.25, 4, 6, and 24 h, and once daily thereafter for a total of 7 days for plasma and PBMC isolation as above. On Study Day 1 (0.5, 0.75, 1, 2, 12, hours) additional blood samples were collected for plasma only. Vaginal and rectal fluid samples were collected on Study Days 1 (6 and 24 h), and 2 to 7. Vaginal and rectal biopsies were collected on Study Days 1, 3, and 7. The samples were processed as above.

All beagle dog studies were non-terminal, and on completion of all in-life procedures, animals were transferred to Sinclair’s Open Colony.

C57BL/6 J Mouse studies were conducted at The Scripps Research Institute using methods described in detail elsewhere [11]. Briefly, seven study groups (*N* = 15 per group) were used as follows: (a) low-releasing TAF implant, single device; (b) low-releasing TAF implant, two devices in one subdermal pocket; (c) low-releasing TAF implant, two devices in separate subdermal pockets; (d) high-releasing TAF implant, single device; (e) high-releasing TAF implant, two devices in one subdermal pocket; (f) high-releasing TAF implant, two devices in separate subdermal pockets; and (g) high-releasing TAF implant, one medicated and one placebo device in one subdermal pocket. For each group, three mice were sacrificed on Study Days 3, 7, 14, 21, and 28 and blood processed to afford plasma and PBMCs as described previously [11].

No additional merino sheep studies were carried out and the sheep data presented here were obtained as part of a previous report [13].

### Bioanalytical

Drug concentrations in plasma (TAF and tenofovir, TFV), PBMCs (tenofovir diphosphate, TFV-DP), rectal and vaginal fluids (TFV), and tissue homogenate (TFV, TFV-DP) samples were measured using liquid chromatography-tandem mass spectrometry (LC–MS/MS) according to methods described in detail elsewhere [10, 14]. Mouse plasma was analyzed at Oak Crest with a lower limit of quantification (LLQ) for TFV in plasma of 5 ng mL^−1^. The remaining samples were analyzed by the Clinical Pharmacology Analytical Laboratory at the Johns Hopkins University School of Medicine with the following LLQs: plasma: TAF, 0.03 ng mL^−1^; TFV, 1 ng mL^−1^; PBMCs: TFV-DP, 5 fmol/sample; vaginal/rectal fluid: TFV, 0.25 ng/sample; vaginal/rectal tissue: TFV, 0.05 ng/sample; TFV-DP, 5 fmol/sample. PBMC results were normalized to the number of cells and reported as fmol/10^6^ cells or as intracellular concentration based on cell volume, in µM, assuming a mean cell volume of 0.2 µL/10^6^ PBMCs [15]. Vaginal/rectal fluid and tissue results were normalized to sample mass and reported as ng mg^−1^ or fmol mg^−1^.

### Pharmacokinetic Model Analyses

Compartmental analyses were performed in Certara’s Phoenix® software (version 8.3, Certara, Princeton, NJ) using a published, simple, structural PK model describing the TFV kinetics [11, 16]. Data were modeled using an extended least-square algorithm with first-order conditional estimation. The systemic parameters, along with the implant *in vivo* release rates on a per animal basis, were used to co-model (and therefore predict) concentration data in dogs during implant dosing.

### Multispecies Comparison of Dose–Response Datasets

Literature datasets linking TAF implant dose (µg kg^−1^ d^−1^) to equilibrium PBMC TFV-DP concentrations were normalized so that all concentrations were expressed as medians and interquartile ranges (IQRs). Some reports presented medians and ranges [17], or medians and standard deviations [18]. Estimation for the reported summary statistics to medians and IQRs was achieved using published approaches [19, 20].

### Data Visualization and Analysis

Post-dose concentrations below the corresponding LLOQ (*C*_*LLQ*_) for inclusion in concentration–time plots and the corresponding summary tables were treated as follows:

1 $$C_{LLQ}=\frac{\mathrm{Assay}\;\mathrm{LLOQ}}2$$

When the assay LLOQ was a function of fluid/tissue mass or PBMC count, the corresponding median quantity (*i.e*., fluid/tissue mass or PBMC count) was included in the denominator [21].

Data were analyzed and plotted in GraphPad Prism (version 9.4.1, GraphPad Software, Inc., La Jolla, CA). Statistical significance is defined as *P* < 0.05. The unpaired, two-tailed, parametric *t*-test with Welch’s correction was used to compare two groups. The paired, two-tailed, nonparametric *t*-test (Wilcoxon matched-pairs signed rank test) was used to compare paired datasets. Graphs were compiled into figures using Adobe Photoshop CS6 (version 13.0, Adobe Systems, Inc., San Jose, CA).

## Results

### *In Vitro *and Multispecies *in Vivo* TAF Delivery Rates

**Fig. 1:**
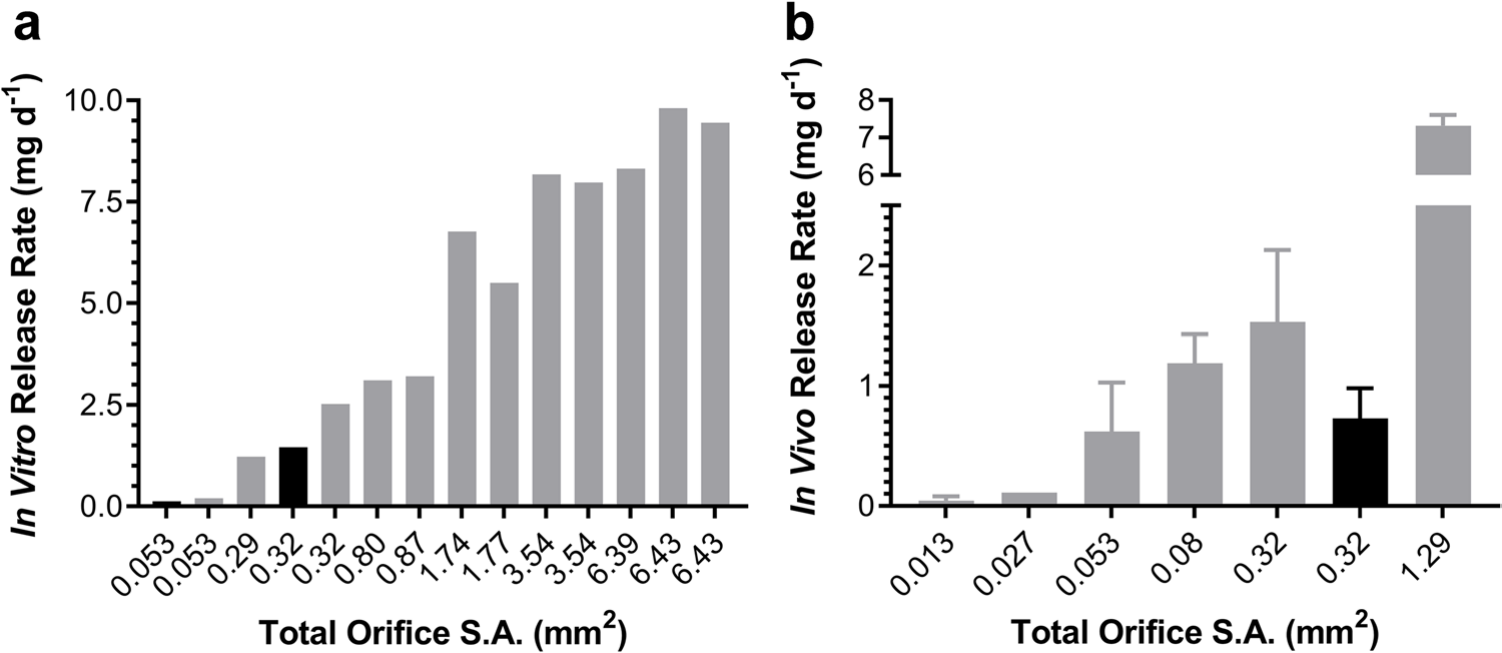
TAF Release Rates Are Tunable as a Function of Total Exposed Implant Orifice Surface Area in 1 cm (black) and 4 cm (gray) Length Implants. (**a**) Compiled *in vitro* data. (**b**) *In vivo* TAF release in beagle dogs.

The parameters determining the TAF release kinetics from our implant platform [10] have been discussed elsewhere [10, 11, 13]. Briefly, drug diffusion occurs exclusively through the delivery orifices fashioned in the silicone implant scaffold, such that the delivery rates are controlled by the diameter and number of channels (*i.e.*, total orifice surface area, S.A.), the properties of any external polymer coating (*i.e.*, implant skin), and possibly implant length. The impact of S.A. on *in vitro* (Fig. 1a) and *in vivo* TAF release rate in beagle dogs (Fig. 1b) was investigated, demonstrating that the drug delivery rates could be tuned over a wide range.

**Fig. 2:**
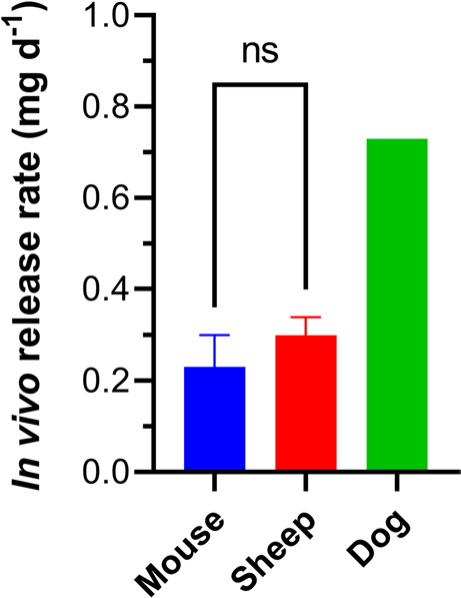
Comparison of TAF *in Vivo* Release Rates (Mean + SEM) from Equivalent Subdermal Implants (length, 10 mm; outer diameter, 2.3 mm; total orifice S.A., 0.32 mm^2^) across Three Animal Species: C57BL/6 J Mice; Merino Sheep; and Beagle Dogs. No error bar is shown for the release rates in dogs as only two animals were used in the study. The mouse and sheep TAF release rates were not significantly different (ns, *P* = 0.4086) when compared using an unpaired, two-tailed *t*-test with Welch’s correction.

Implants (length, 10 mm; outer diameter, 2.3 mm; total orifice S.A., 0.32 mm^2^) were evaluated in three species (Fig. 2). The *in vivo* TAF release rates (mean + SEM) rates were: C57BL/6 J mice, 0.23 ± 0.07 mg d^−1^ [13]; merino sheep, 0.30 ± 0.04 mg d^−1^ [13]; and beagle dogs, 0.73 mg d^−1^. Standard errors on the mean in dogs could not be calculated as only two animals were used in the study (individual release rates, 0.55 and 0.90 mg d^−1^). The small group size used in the dog implant study also precluded the comparison of the groups in Fig. 2 using a *t*-test, as we did not have the statistical power to detect a significant difference between the datasets. However, the mean TAF release rates in dogs were 3.2 and 2.4 times higher than the corresponding values in mice and sheep, respectively. The *in vivo* TAF release rates in mice and sheep were not statistically significantly different (*P* = 0.4086) using an unpaired, two-tailed *t*-test with Welch’s correction (Fig. 2).

The impact of TAF implant characteristics on *in vitro* and *in vivo* drug release rates, and the associated *in vitro-in vivo* correlation (IVIVC) is shown in Table I. While the IVIVC was variable, it was highest in dogs, driven by the higher *in vivo* TAF release rates compared to the other two species.

**Table I:** Impact of Implant Formulation Parameters on Drug Release Kinetics (Mean ± SEM) across Animal Species

Species	Implant Length (mm)	S.A.^a^ (mm^2^)	*In Vitro* Release Rate (mg d^−1^)	*In Vivo* Release Rate (mg d^−1^)	IVIVC^b^
Dog	40	0.32	2.53 ± 0.08	1.53 ± 0.65	0.60
Dog	40	1.29	4.80^c^	7.32 ± 0.29	1.53
Dog	10	0.32	1.46 ± 0.03	0.73 ± 0.25	0.50
Mouse	10	0.32	1.46 ± 0.03	0.23 ± 0.03	0.16
Sheep	10	0.32	1.46 ± 0.03	0.30 ± 0.08	0.21

^a^S.A. is total orifice surface area exposed per implant, calculated as S.A. = *n* π (*d*/2)^2^, where *n* is number of orifices and *d* is orifice diameter

^b^IVIVC, *in vitro*-*in vivo* correlation, calculated by dividing the *in vitro* release rate by the *in vivo* release rate for a given formulation

^c^Estimated by interpolation from *in vitro* release rate *versus* S.A. data

### Multiple Implants in Beagle Dogs Result in Unexpected Pharmacokinetics

**Fig. 3:**
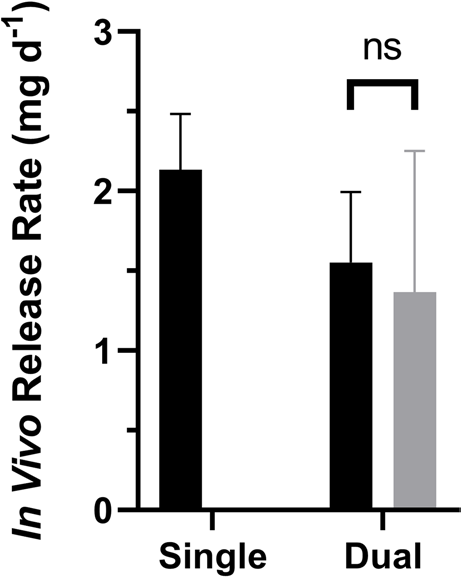
Comparison of TAF *in Vivo* Release Rates (Mean + SD) from Subdermal Implants (Length, 40 mm; Outer Diameter, 2.3 mm) in Beagle Dogs over 30 Days Estimated via Residual Drug Analysis in Used Devices on Day 30. Single, one implant (*N* = 3; one implant from the fourth animal could not be recovered); dual, two implants in the same subcutaneous pocket (*N* = 8). The TAF release rates for implants in the same subcutaneous pocket were not significantly different (ns, *P* = 0.8125) when compared using a paired, two-tailed *t*-test (Wilcoxon matched-pairs signed rank test).

Implants with high TAF delivery rates (Fig. 3, Table II) were evaluated in beagle dogs using two groups consisting of: (a) single implants (*N* = 4); and (b) two implants in one subcutaneous pocket (*N* = 8). The per-implant *in vivo* TAF release rate parameters are summarized in Fig. 3 and Table II.

**Table II:** *In Vivo* TAF Release Rates for Human-sized Subdermal Implants in Beagle Dogs

Implants per Subcutaneous Pocket	Number of Animals	*In Vivo* Release Rate, Median, IQR (mg d^−1^)	*In Vivo* Release Rate,^b^ Median, IQR (µg kg^−1^ d^−1^)
1	3^a^	2.07, 1.95–2.29	199, 191–256
2	8	2.89, 2.25–3.50^c^	268, 235–378

^a^ Four animals were used, but the implant from one of the dogs could not be recovered

^b^ Body weight adjusted

^c^ Sum of both implants

**Fig. 4:**
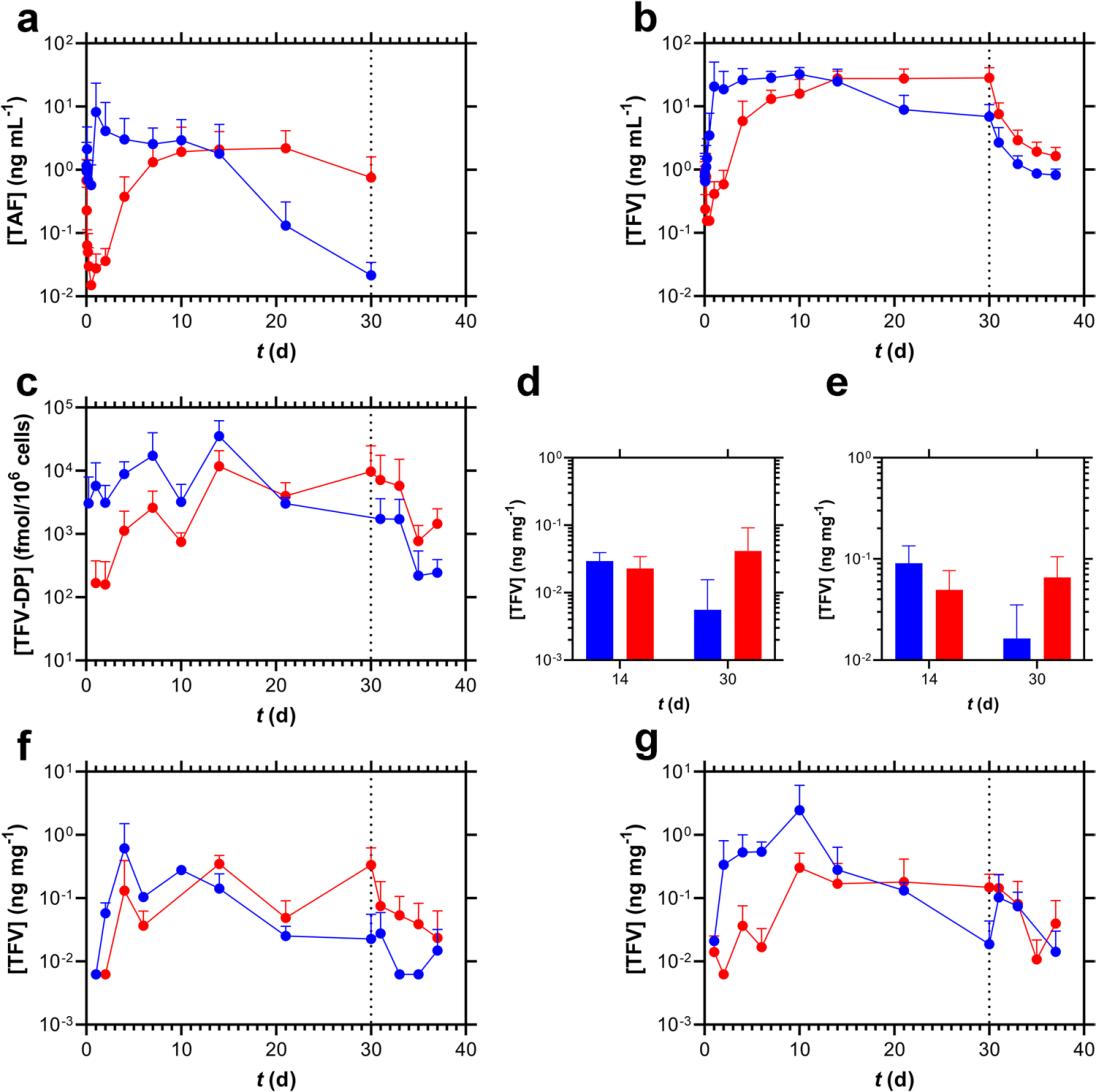
Subdermal Placement of TAF Implants in Beagle Dogs Results in Differing Concentration–time Profiles Depending on the Number of Devices per Subdermal Pocket; blue, one implant per pocket (*N* = 4); red, two implants per pocket (*N* = 8); data represent means + SD; broken vertical line, time of implant removal. (**a**) Plasma TAF. (**b**) Plasma TFV. (**c**) PBMC TFV-DP. (**d**) Vaginal tissue TFV. (**e**) Rectal tissue TFV. (**f**) Vaginal fluid TFV. (**g**) Rectal fluid TFV.

The concentration–time profiles associated with the above implant use are shown in Fig. 4 and summarized in Table III. While TAF and its key metabolites, TFV and TFV-DP, in systemic compartments rapidly reached plateau concentrations in the single implant group (blue plots; Fig. 4a, plasma TAF; Fig. 4b, plasma TFV; Fig. 4c, PBMC TFV-DP), the dual implant group exhibited a 14-day lag (red plots; Fig. 4a, plasma TAF; Fig. 4b, plasma TFV; Fig. 4c, PBMC TFV-DP).

**Table III:** Summary of TAF, TFV, and TFV-DP Concentrations Measured in Beagle Dogs Following TAF Delivery from Subdermal Implants. The Calculations are based on Measurements During the Analyte Concentration Plateau (see Fig. 4)

Implant configuration, analyte, matrix,^a^ units	*n*	% above LLOQ^b^	Analysis window study days	Median (IQR^c^)
Single implant, TAF, plasma, ng mL^−1^	24	92	1–14	1.28 (0.222–3.24)
Dual implants, TAF, plasma, ng mL^−1^	16	100	14–21	1.78 (0.492–3.33)
Single implant, TFV, plasma, ng mL^−1^	24	96	1–14	24.7 (17.1–35.9)
Dual implants, TFV, plasma, ng mL^−1^	24	100	14–30	25.9 (20.6–35.5)
Single implant, TFV-DP, PBMCs, fmol/10^6^ cells	40	95	0.25–21	3.90 × 10^3^ (1.95 × 10^3^–9.92 × 10^3^)
Dual implants, TFV-DP, PBMCs, fmol/10^6^ cells	24	100	14–30	5.78 × 10^3^ (2.91 × 10^3^–8.43 × 10^3^)
Single implant, TFV, vaginal tissue, ng mg^−1^	8	63	14–30	2.06 × 10^–2^ (5.95 × 10^–4^-2.55 × 10^–2^)
Dual implant, TFV, vaginal tissue, ng mg^−1^	16	88	14–30	2.31 × 10^–2^ (1.39 × 10^–2^-3.18 × 10^–2^)
Single implant, TFV, rectal tissue, ng mg^−1^	8	75	14–30	3.78 × 10^–2^ (2.12 × 10^–2^-8.27 × 10^–2^)
Dual implant, TFV, rectal tissue, ng mg^−1^	16	100	14–30	4.90 × 10^–2^ (2.82 × 10^–2^-8.09 × 10^–2^)
Single implant, TFV, vaginal fluid, ng mg^−1^	17	82	4–30	9.62 × 10^–2^ (2.54 × 10^–2^-0.183)
Dual implant, TFV, vaginal fluid, ng mg^−1^	27	89	4–30	9.09 × 10^–2^ (2.88 × 10^–2^-0.236)
Single implant, TFV, rectal fluid, ng mg^−1^	20	80	4–30	0.186 (5.58 × 10^–2^-0.467)
Dual implant, TFV, rectal fluid, ng mg^−1^	40	83	4–30	6.60 × 10^–2^ (1.99 × 10^–2^-0.187)

^a^ All values correspond to timepoints with the implant in place

^b^ Proportion of samples that contained quantifiable drug concentrations

^c^ Interquartile range, between first (25^th^ percentile) and third (75^th^ percentile) quartiles

### Multiple Implant Studies in C57BL/6 J Mice

**Fig. 5:**
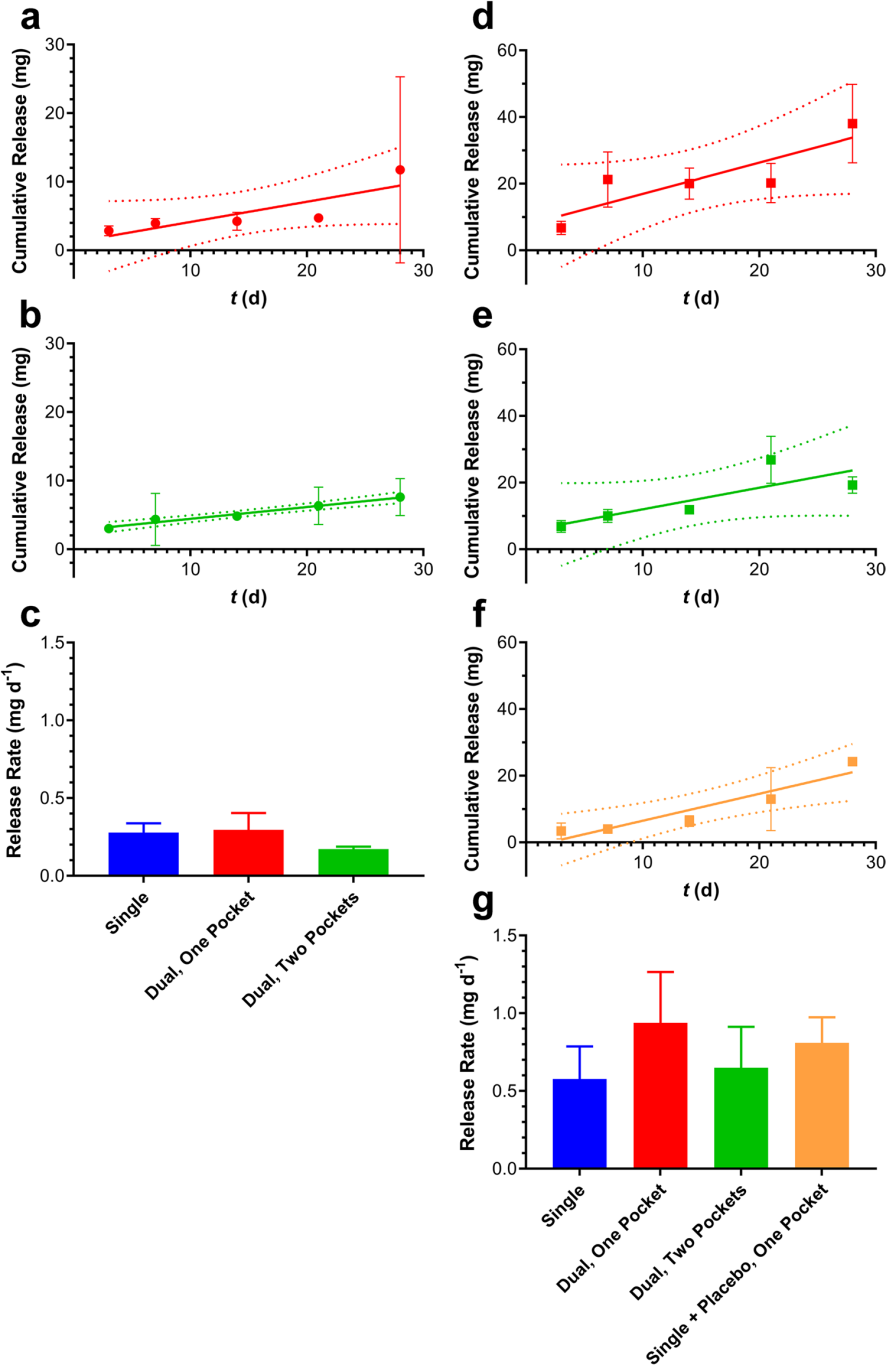
Comparison of TAF *in Vivo* Release Rates from Subdermal Implants (length, 10 mm; outer diameter, 2.4 mm) in C57BL/6 J Mice (*N* = 3 per timepoint) over 28 Days Estimated via Residual Drug Analysis in Used Devices at Every Timepoint; means ± SEM; (**a, b, d, e, f**) solid line, simple linear regression; broken lines, 95% confidence bands; circles, low TAF releasing implant configuration; squares, high TAF releasing implant configuration. *In vivo* drug release plots for single implant groups have been reported elsewhere [11]. One or both implants were missing at collection for: low, dual, one pocket (D21); high, dual, one pocket (D28); high, dual, separate pockets (D14, two animals; D21; D28). (**a**) Low-releasing, two implants in one subcutaneous pocket. (**b**) Low-releasing, two implants in separate subcutaneous pockets. (**c**) Comparison of *in vivo* TAF release rates for low-releasing implants as a function of configuration. (**d**) High-releasing, two implants in one subcutaneous pocket. (**e**) High-releasing, two implants in separate subcutaneous pockets. (**f**) High-releasing, one medicated and one placebo implant in one subcutaneous pocket. (**g**) Comparison of *in vivo* TAF release rates for high-releasing implants as a function of configuration.

The unexpected slow rise to equilibrium observed for systemic analyte concentrations in the beagle dog study group when two TAF implants were placed in one subcutaneous pocket led us to conduct a complementary study in C57BL/6 J mice. The study was made up of seven groups (*N* = 15 per group, Fig. 5): (a) low-releasing TAF implants, one implant, described in detail elsewhere [11]; (b) low-releasing TAF implants, two implants in one subcutaneous pocket; (c) low-releasing TAF implants, two implants in separate subcutaneous pockets; (d) high-releasing TAF implants, one implant, described in detail elsewhere [11]; (e) high-releasing TAF implants, two implants in one subcutaneous pocket; (f) high-releasing TAF implants, two implants in separate subcutaneous pockets; and (g) high-releasing TAF implants, one medicated and one placebo implant in one subcutaneous pocket. The associated *in vivo* TAF release rates are summarized in Fig. 5. As in our previous report [11], animals were sacrificed at every sample collection timepoint, allowing the *in vivo* TAF release profiles to be determined empirically for all implant groups, demonstrating that zero order (linear) kinetics have been achieved (Fig. 5a, b, d, e, f). When multiple implants per animal were used, the cumulative TAF release was calculated as the sum of the two implants (*i.e.*, sum of per implant TAF released over the corresponding time period). The *in vivo* TAF release rates for low- (Fig. 5c) and high-releasing (Fig. 5g) implants follow similar trends. The highest release rates were observed with both implants in the same pocket, although these were not double the corresponding single implant groups. Surprisingly, when two devices were implanted in separate subcutaneous pockets, the total TAF release rates were lower or comparable to the single implant groups. When high-releasing implants were collocated with placebo implants, the *in vivo* release rates were greater than for the corresponding single implants (Fig. 5g).

**Fig. 6:**
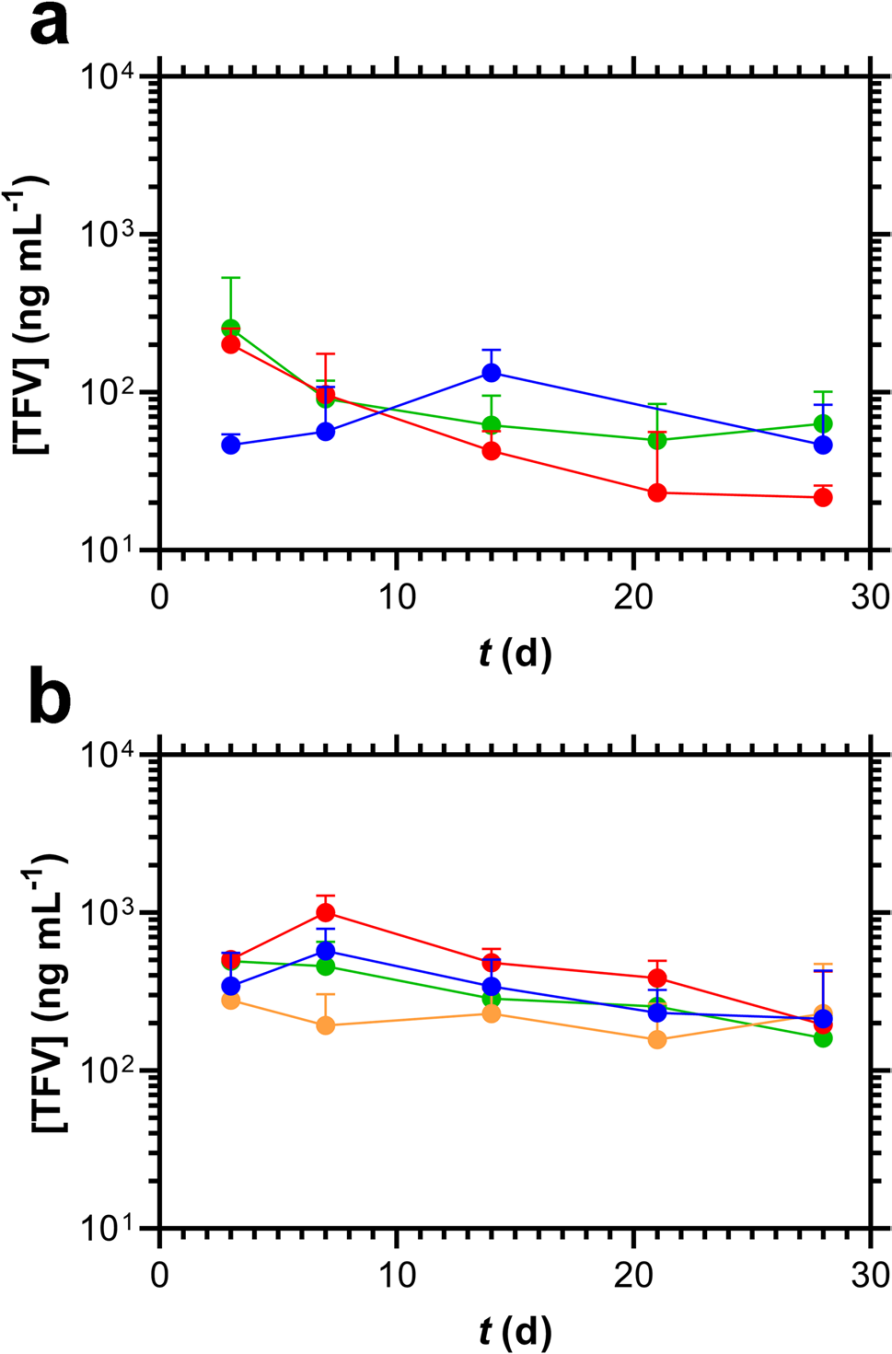
Plasma TFV Concentration–time Profiles (Mean + SD) in C57BL/6 J mice (*N* = 3 per Timepoint) for Low- (**a**) and High-releasing (**b**) Implants. (**a**) Blue, single implant; red, two implants, one pocket; green, two implants, separate pockets. (**b**) Blue, single implant; red, two implants, one pocket; green, two implants, separate pockets; orange, one medicated and one placebo implant, one pocket. One or both implants were missing at the time of collection for: low, dual, one pocket (D21); high, dual, one pocket (D28); high, dual, separate pockets (D14, two animals; D21; D28).

The plasma TFV concentration–time profiles corresponding to the low- and high-releasing TAF implant groups described in Fig. 5 are presented below in Fig. 6. No obvious trends linking *in vivo* TAF release rate and TFV plasma concentrations were apparent (see also Table IV). However, no extended (*i.e.*, 2-week) TFV plasma concentration rise to *C*_*max*_ was observed in mice when two implants were placed in the same subcutaneous pocket. These results contrast sharply to the above dog data (Fig. 4b).

**Table IV:** Summary of Plasma TFV Concentrations Measured in C57BL/6 J Mice Following TAF Delivery from Subdermal Implants over 28 Days (see Fig. 6)

Implant configuration^a^	*n*	% above LLOQ^b^	Plasma [TFV] (ng mL^−1^) Median (IQR^c^)
Low releasing, single implant	12	83	59.4 (45.9–81.7)
Low releasing, dual implants, one pocket	15	87	39.0 (24.0–113)
Low releasing, dual implants, separate pockets	15	100	77.0 (42.6–94.6)
High releasing, single implant	15	100	307 (193–487)
High releasing, dual implants, one pocket	15	93	484 (400–569)
High releasing, dual implants, separate pockets	15	100	299 (214–426)
High releasing, one medicated and one placebo implant, one pocket	15	93	200 (129–279)

^a^ All values correspond to timepoints with the implant in place

^b^ Proportion of samples that contained quantifiable drug concentrations

^C^ Interquartile range, between first (25^th^ percentile) and third (75^th^ percentile) quartiles

### Intravenous TAF Pharmacokinetics in Beagle Dogs

**Fig. 7:**
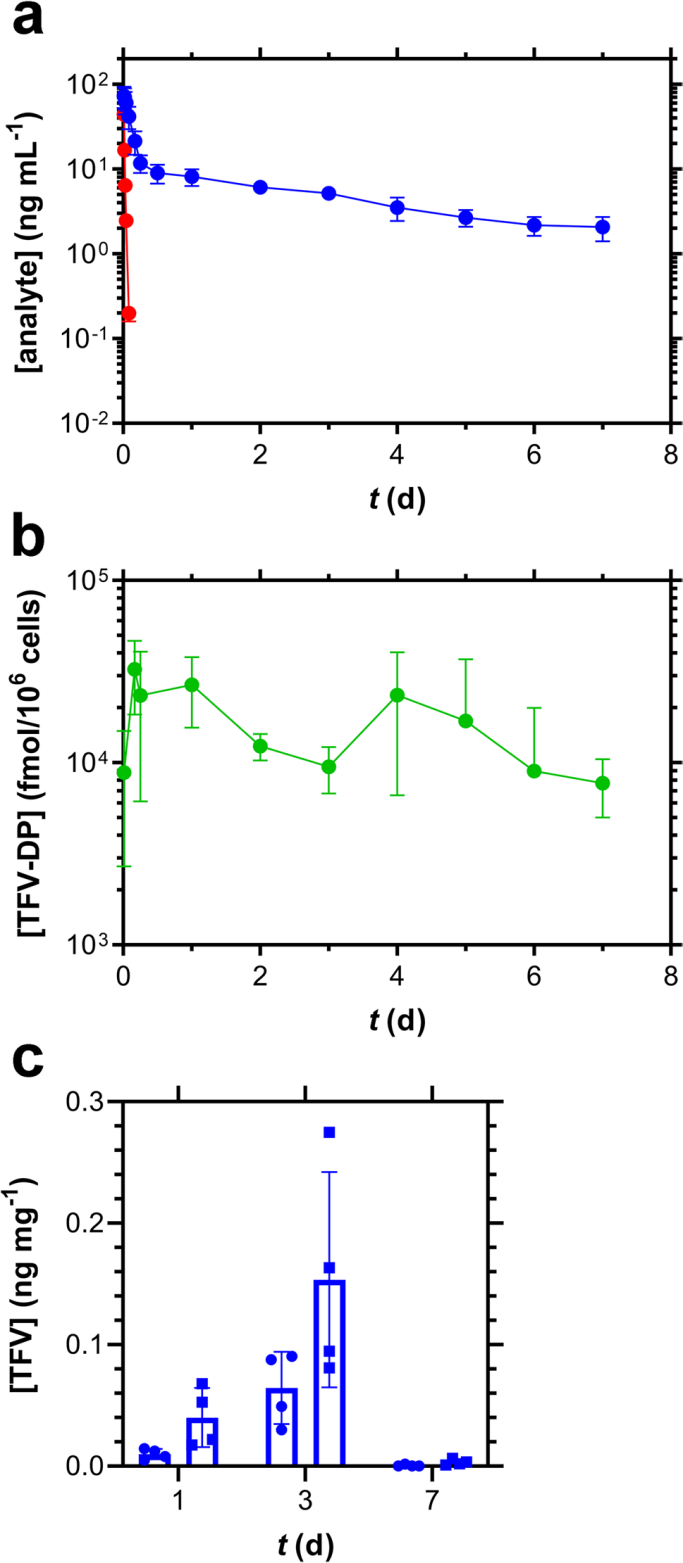
Pharmacokinetics of IV TAF Injection (1.0 mg kg^−1^) in Beagle Dogs (*N* = 4). (**a**) Plasma TAF (red) and TFV (blue) concentration–time profiles. (**b**) PBMC TFV-DP concentration–time profiles. (**c**) Vaginal (circles) and rectal (squares) tissue homogenate TFV concentrations at three timepoints spanning 7 days post IV injection.

Intravenous (IV), bolus TAF dosing in beagle dogs, followed by the measurement concentration–time profiles of TAF and its metabolites in key anatomic compartments (Fig. 7) was performed to enable PK modeling in an effort to explain the above results. Plasma TAF and TFV as well as PBMC TFV-DP concentration–time profiles followed typical monoexponential and biexponential decay, respectively, following IV TAF injection (Fig. 7a and b). The biphasic pattern observed on the logarithm of PBMC TFV-DP concentration–time curve, with concentration maxima at 24 and 96 h (Fig. 7b), also was observed in women [22], and attributed to complicated TFV intracellular phosphorylation.

An existing TAF PK model [16] was adapted to estimate TAF systemic PK parameters in dogs by simultaneously modeling TFV and TFV-DP concentrations following IV TAF injection and implant dosing, analogous to the approach we used previously in C57BL/6 J mice [11]. As in our mouse PK modeling studies, there was no indication of flip-flop kinetics, evidenced by parallel terminal (*i.e.*, elimination) phases over the final timepoints. The results of the simultaneous model (co-model) describing the systemic parameters for TFV are presented in Table V. The elimination half-life, *t*_*1/2*_, for TFV in plasma was 70 h, longer than the value of 53 h observed in mice [11], and the contribution of the non-linear Michaelis–Menten intrinsic clearance was found to be negligible (*V*_*max*_*/K*_*m*_ = 1.7 × 10^–4^ L d^−1^). For TFV-DP in PBMCs, *t*_*1/2*_ was found to be 10 h, shorter than in mice (41 h) [11] and humans (48 h) [22].

**Table V:** Model Describing the Systemic Pharmacokinetic Parameters for Plasma TFV in Beagle Dogs, Using IV and Implant Co-modeling across Multiple Studies (*i.e.*, our 2014 study [10] as well as Studies S14161 and S14168 Combined)

Parameter	Units	Estimate	CV (%)
*V*	L	84.7	16.5
*Cl*	L d^−1^	174	11.4
*V*_*p*_	L	534	18.5
*Cl*_*p*_	L d^−1^	681	24.7
*t*_*1/2*_	h	70	NA
MAT	min	< 1	25.8
*F*		3.4	25.6
*K*_*out*_	d^−1^	1.84	77.6
*V*_*m*_*/K*_*m*_	L d^−1^	1.7 × 10^–4^	80.7
Model error			
Plasma	%	74.6	11.1
PBMC	%	105	12.2

*V*, volume of distribution of the central compartment; *Cl*, total body clearance; *V*_*p*_, volume of distribution of the second compartment; *Cl*_*p*_, clearance from the second compartment; *t*_*1/2*_, elimination half-life; MAT, mean absorption time from the subcutaneous pocket; *F*, overall bioavailability across all studies; *K*_*out*_, elimination rate parameter; *V*_*m*_, maximum rate achieved by the system using Michaelis–Menten kinetics; *K*_*m*_, Michaelis constant; Model Error, multiplicative error that is proportional to concentration; NA, not applicable as this parameter was not estimated by the model, but is derived from other model parameters

### Modeling TAF Implant PK in Beagle Dogs and Simulation of Concentration–time Profiles

Pharmacokinetic parameters for TAF metabolites corresponding to TAF implant studies in beagle dogs were derived with our simultaneous model (co-model) using IV and implant systemic concentrations. The results are presented in Table VI and represent a number of key findings:

- The rate of conversion of plasma TFV to PBMC TFV-DP appeared more efficient following IV dosing (2,030 × 10^–6^ L d^−1^) compared to implant dosing (mean range, 10.6–378 × 10^–6^ L d^−1^),
- The rate of conversion of plasma TFV to PBMC TFV-DP from the current dog implant study (Study S14168) appeared more efficient (mean range: 185–378 × 10^–6^ L d^−1^) compared to our previous, 2014 study [10] using TAF implants in beagle dogs (10.6 × 10^–6^ L d^−1^),
- The rate of conversion of plasma TFV to PBMC TFV-DP in the dog TAF implant Study S14168 appeared more efficient when implants were in a single pocket (378 × 10^–6^ L d^−1^) compared two pockets (185 × 10^–6^ L d^−1^),
- The apparent TAF bioavailability (*F*) in dog Study S14168 appeared greater when a single implant was used (9.62) compared when two implants were located in one subcutaneous pocket (1.54),
- When only one TAF implant was used in beagle dogs, the apparent TAF bioavailability was similar in Study S14168 (9.62) compared to our previous report (11.2) [10],
- In all cases when TAF was delivered via subdermal implant, the apparent bioavailability was greater than unity, the value ascribed to IV dosing.

**Table VI:** Pharmacokinetic Parameters for Plasma TFV and PBMC TFV-DP in Beagle Dogs, Derived Using IV and Implant Co-modeling

	S14161^a^	2014^b^	S14168^a^		Overall	
	IV	1 Pocket	1 Pocket	2 Pockets	1 Pocket	2 Pockets
	*N* = 4	*N* = 4	*N* = 4	*N* = 8	*N* = 8	*N* = 8
*F*						
Mean (CV, %)	NA	11.2 (30.6)	9.62 (124)	1.54 (20.3)	10.4 (78.4)	1.54 (20.3)
Median (min, max)	NA	9.82 (8.90, 16.3)	4.78 (1.81, 27.1)	1.47 (1.22, 1.99)	9.28 (1.81, 27.1)	1.47 (1.22, 1.99)
*V*_*m*_*/K*_*m*_ (10^6^ L d^−1^)						
Mean (CV, %)	2030 (34.0)	10.6 (57.8)	378 (46.4)	185 (79.4)	194 (117)	185 (79.4)
Median (min, max)	2020 (1190, 2880)	10.0 (4.7, 17.9)	422 (128, 539)	139 (101, 545)	72.9 (4.7, 539)	139 (101, 545)

^a^ Study number

^b^ Historical TAF implant study [10]

^c^ Not applicable

**Fig. 8:**
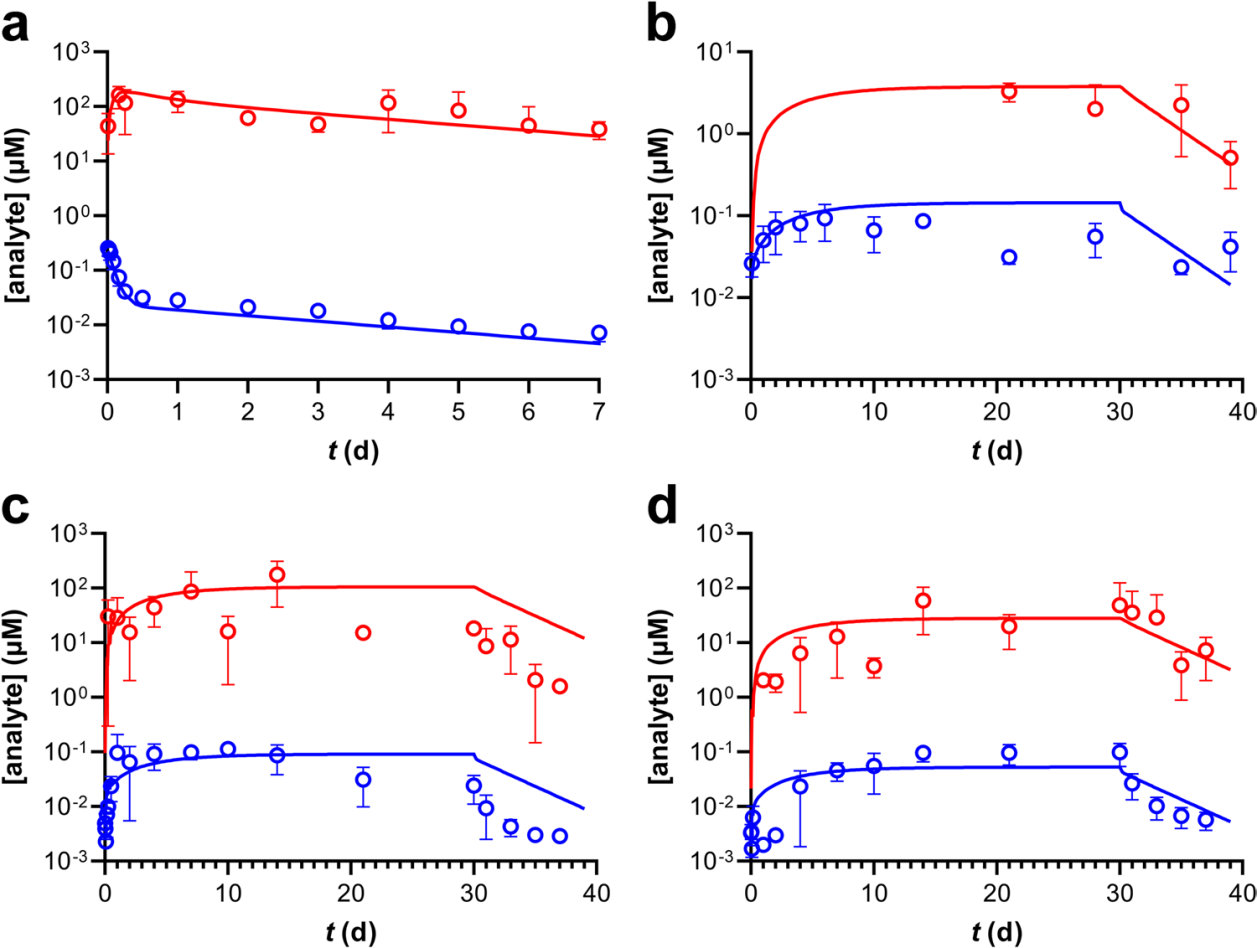
Pharmacokinetic Simulations (Solid Lines) of Plasma TFV (blue) and PBMC TFV-DP (red) Concentrations in Beagle Dogs Based on Actual *in Vivo* TAF Implant Release Characteristics and the Co-model Parameters from Table V and VI. Circles represent mean (± *SD*) measured drug concentrations, and all concentration units are represented in micromolar (µM) for ease of comparison. (**a**) IV study (S14161, see Fig. 7). (**b**) Historical single TAF implant study (*in vivo* release rate, 1.07 mg d^−1^) [10]. (**c**) Single implant, present study (S14168, see Fig. 4). (**d**) Two implants in one subcutaneous pocket, present study (S14168, see Fig. 4). For (**c**) and (**d**), implants were removed on Study Day 30.

The PK model and parameters (Table V and VI) were employed to simulate systemic drug exposure in beagle dogs following TAF delivery from subcutaneous implants (Fig. 8), utilizing only the device *in vivo* release rates as inputs (Fig. 3). The data were converted to molar concentrations, as described previously [11], to allow both analytes (TFV and TFV-DP) to be visualized on the same *y*-axis, even though they were measured in different anatomic compartments. The following conversions from commonly reported units are included for the sake of convenience: plasma TFV, 1.0 ng mL^−1^ = 3.5 × 10^–3^ µM (3.5 nM); PBMC TFV-DP, 1.0 fmol/10^6^ cells = 5.0 × 10^–3^ µM (5.0 nM). The experimental observations (circles, means ± SD) are equivalent to those shown in Fig. 4 and Fig. 7. Overall, using the apparent bioavailability shown in Table VI, there was agreement between model simulations and observed data, even after the implants were removed. For Fig. 8b, the devices were not removed until Study Day 40, but the TAF implant reservoir was becoming depleted around Study Day 30, accounting for the decreasing analyte concentrations thereafter.

## Discussion

The primary goal of the current report was to compare preclinical models with wide range of body weights for the evaluation of *in vivo* drug release kinetics and concomitant PKs using our subdermal TAF implant system [10]. While safety endpoints, particularly local tolerance, represent a critically important component in the successful development of a subdermal TAF implant, they are not a focus here, and have been discussed in detail previously [8, 10, 11, 13]. In summary, in the three species studied (mice, dogs, and sheep), we did not observe concerning local toxicity related to the drug or device, other than the expected foreign body response. However, there are some caveats associated with this conclusion. Because our focus has been on developing the implant system for controlled, linear TAF delivery, the preclinical studies have been 40 days, or shorter. Longer studies are envisioned in the future with lead implant candidates. Implants with *in vivo* TAF release rates in excess of 1 mg d^−1^ did exhibit dose-dependent local toxicity in beagle dogs [8], but these are higher than the anticipated dose required to achieve prophylactic efficacy against HIV-1 infection in humans [10, 23].

### *In Vitro *and *In Vivo* TAF Release Kinetics from Implant Prototypes

Our subdermal implant platform involves a drug-impermeable cylindrical silicone reservoir perforated with one or more orifices for drug delivery [10]. The total orifice surface area (S.A.) determined the control of *in vitro* TAF release rates in the 0.13 to 9.8 mg d^−1^ range (Fig. 1a), with release exhibiting zero order (linear) kinetics [10]. As in our previous report [11], we leveraged the numerical power afforded by the mouse model to show that the implants maintained linear TAF release kinetics *in vivo* (Fig. 5a, b, d-f). The relationship between total implant orifice S.A. and TAF release rate translated to beagle dogs (Fig. 1b), although the corresponding *in vivo* release was lower than *in vitro* (Fig. 1, Table I). Mouse-sized implants (length, 10 mm) had lower *in vitro* and *in vivo* release rates than human-sized implants (length, 40 mm) with the same orifice configuration (Fig. 1), an observation that was surprising as both devices have the same exposed orifice S.A. In the longer implants, the drug diffusion distance to the orifices is greater and that would have led to slower, not faster release kinetics.

When comparing the *in vivo* TAF release kinetics of implants with the same configuration across three animal species, there was no significant difference (*P* = 0.4086) in release rate between C57BL/6 J mice and merino sheep (Fig. 2), the extremes on the body weight spectrum, but the release rate in beagle dogs was up to 3 times higher, an unexpected result. The IVIVC consistently was found to be larger in dogs than in mice and sheep (Table I), and deviated from unity. It is not unexpected to observe non-ideal IVIVC behavior with our implant systems, probably due to the mode of drug delivery. TAF Is delivered via orifices that act as point sources rather than through the entire surface of the device, as with other resorbable [24] and non-resorbable [17] reservoir-style implants where the entire, unperforated outer shell acts as a sustained-release membrane. Our *in vitro* system clearly does not recapitulate the complexities encountered during *in vivo* implant TAF delivery. However, successful evaluation of implant prototypes *in vitro* to guide product development, and as a quality control tool, is not predicated by an IVIVC of unity.

### The Pharmacokinetics Associated with Placing Multiple Implants in One Subdermal Pocket

The simultaneous use of multiple implants to increase duration between placement/removal procedures has been commonplace for contraceptive implants for 40 years (NORPLANT levonorgestrel implant system comprised of six silicone rods). It is possible that multiple implants also will be used for HIV PrEP. When two paired TAF implants were placed in one subcutaneous pocket in beagle dogs, the individual devices delivered TAF at rates that were not statistically different (*P* = 0.8125). The total drug released in both groups (*i.e.*, single and dual implant groups) was not equivalent (Fig. 3, Table II). Unexpectedly, the dual implant group exhibited a slow (*ca*. two-week) rise to *C*_*max*_ for plasma TAF and TFV, as well as PBMC TFV-DP concentrations, not observed in the single implant group (Fig. 4a-c). This finding prompted us to further investigate the effect in C57BL/6 J mice, as summarized in Fig. 5. We used plasma TFV concentrations to investigate whether the dual implant effect observed in dogs translated to mice, and found that the rise to *C*_*max*_ was independent of the number of implants or where they were placed, and no two-week creep was observed. It appears that the unexplained slow rise of TAF and metabolite systemic concentrations is unique to dogs. The dual implant effect also was not observed by Gatto *et al*. in New Zealand white rabbits or rhesus macaques using a different TAF implant technology [24].

### TAF Implant Pharmacokinetic Modeling in Beagle Dogs

A TAF IV (bolus) dosing study in beagle dogs (Fig. 7) was carried out to derive the associated systemic PK parameters (Table V), as these were not available in the literature. We previously used an analogous approach in C57BL/6 J mice [11], and a PK model adapted from the literature [16]. We subsequently co-modeled the TAF IV and implant data (Table VI) and simulated the systemic drug metabolite exposure (plasma TFV and PBMC TFV-DP, Fig. 8) based solely on *in vivo* implant release rates using data from this study and our first report in beagle dogs [10]. The model performed well and allowed us to answer a number of mechanistic questions, as described above (see Results).

The relationship between long-acting, subcutaneous TAF dosing via implant and the concomitant systemic, metabolite concentration–time profiles is complex, as discussed in detail elsewhere [11]. A number of possible non-linear processes are involved starting at the subcutaneous dosing site, and proceeding to other anatomic compartments as TAF is cleared by migration (via passive diffusion and possibly active transport) to afford the active anabolite, TFV-DP, in immune cells capable of supporting HIV-1 replication. Because these processes are rapid and the many intermediates unstable, the nature of these non-linear processes can be challenging to study. Pharmacokinetic modeling in dogs resulted in the important finding that the apparent TAF bioavailability (*F*) from single subdermal implants was *ca*. 10 × higher than when TAF was administered by IV injection (Table VI). The increase in apparent *F* during low, continuous TAF delivery compared to high, bolus dosing is consistent with our prior findings in mice, where *F*(TAF) from single subdermal implants was 4–5 × higher than when TAF was administered by IV or subcutaneous injection [11]. Our observation in two species that the apparent TAF bioavailability is 4–tenfold higher when administered via subcutaneous implant *(i.e.*, sustained release) compared to bolus dosing suggests the physiologic phenomenon is real and could translate to humans. The underlying complex absorption patterns need to be considered in any animal-to-human extrapolation of exposure following implant administration, via a number of hypothesized processes (*e.g.*, saturation of molecular transporters and metabolizing enzymes during bolus, but not long-acting dosing) discussed previously [11]. The initial bioconversion from TAF to TFV also is not conserved across species [25]. In addition, the possibility of changes in gene expression induced by TAF and its metabolites –either by pulsatile, bolus or extended, continuous administration– needs to be explored. For example, García-Lerma *et al*. found that repeated oral administration of high TAF doses in rhesus macaques led to an increase of deoxyadenosine triphosphate (dATP) concentrations in a compartment-selective manner (*i.e.*, concentrations in rectal lymphocytes were *ca*. 100-fold higher than in circulating lymphocytes and lymphoid tissue) [26]. The intended function of TFV-DP is to compete with the natural nucleotide, dATP, as a substrate for HIV-1 reverse transcriptase.

Our TAF implant PK modeling in beagle dogs yielded another important finding. When two implants were placed in one subcutaneous pocket, the apparent TAF bioavailability went from 9.6 (single implant in dogs) to 1.5 (Table VI), only slightly higher than with IV bolus dosing. The result indicates that two, high-releasing TAF implants placed together act similarly to bolus systemic dosing in terms of blocking or inhibiting certain processes responsible for increasing drug anabolite exposure.

### Dose–Response Comparisons Across Preclinical Models and Implant Technologies

There are few ARV drugs available to the research community with sufficient potency for viable delivery from subdermal implants (*i.e.*, duration ≥ 6 months). Islatravir (ISL) is a highly potent nucleoside reverse transcriptase translocation inhibitor with a long intracellular half-life. Results from a phase 1 trial evaluating a subdermal implant delivering ISL provided encouraging PK and safety outcomes [27]. However, on Dec. 13, 2021, the US FDA placed clinical holds on all trials involving ISL due to decreases in total lymphocyte and CD4^+^ T-cell counts in some participants [28] that need to be resolved. The potent capsid inhibitor lenacapavir is being developed for long-acting dosing by Gilead Sciences and currently is not available for external product development. An extended-release, injectable formulation of the integrase strand transfer inhibitor cabotegravir (CAB) has demonstrated superiority over daily oral tenofovir disoproxil fumarate-emtricitabine in the prevention of HIV-1 [29, 30]. Consequently, the CAB nanosuspension, administered as an IM injection (600 mg in 3 mL) once every two months, was approved by the US FDA (Dec. 21, 2021) for HIV-1 PrEP [31]. However, the high CAB protein binding may prohibit practical, long-term (≥ 6 months) delivery via subdermal implant. Karunakaran *et al*. showed that four implants [32], consisting of CAB pellets sealed in a hydrophilic poly(ether-urethane) tube, were needed to maintain plasma drug concentrations associated with *ca*. 97% protection in rhesus macaques (1 × protein-adjusted IC_90_, PA-IC_90_, 166 ng mL^−1^) [33, 34], while even six implants could not maintain median plasma CAB concentrations above the 4 × PA-IC_90_, a common preclinical target. The limited potent, available ARV drug landscape has led us [10, 11, 13] and others [8, 17, 24, 35–40] to develop complementary TAF implant technologies.

**Fig. 9:**
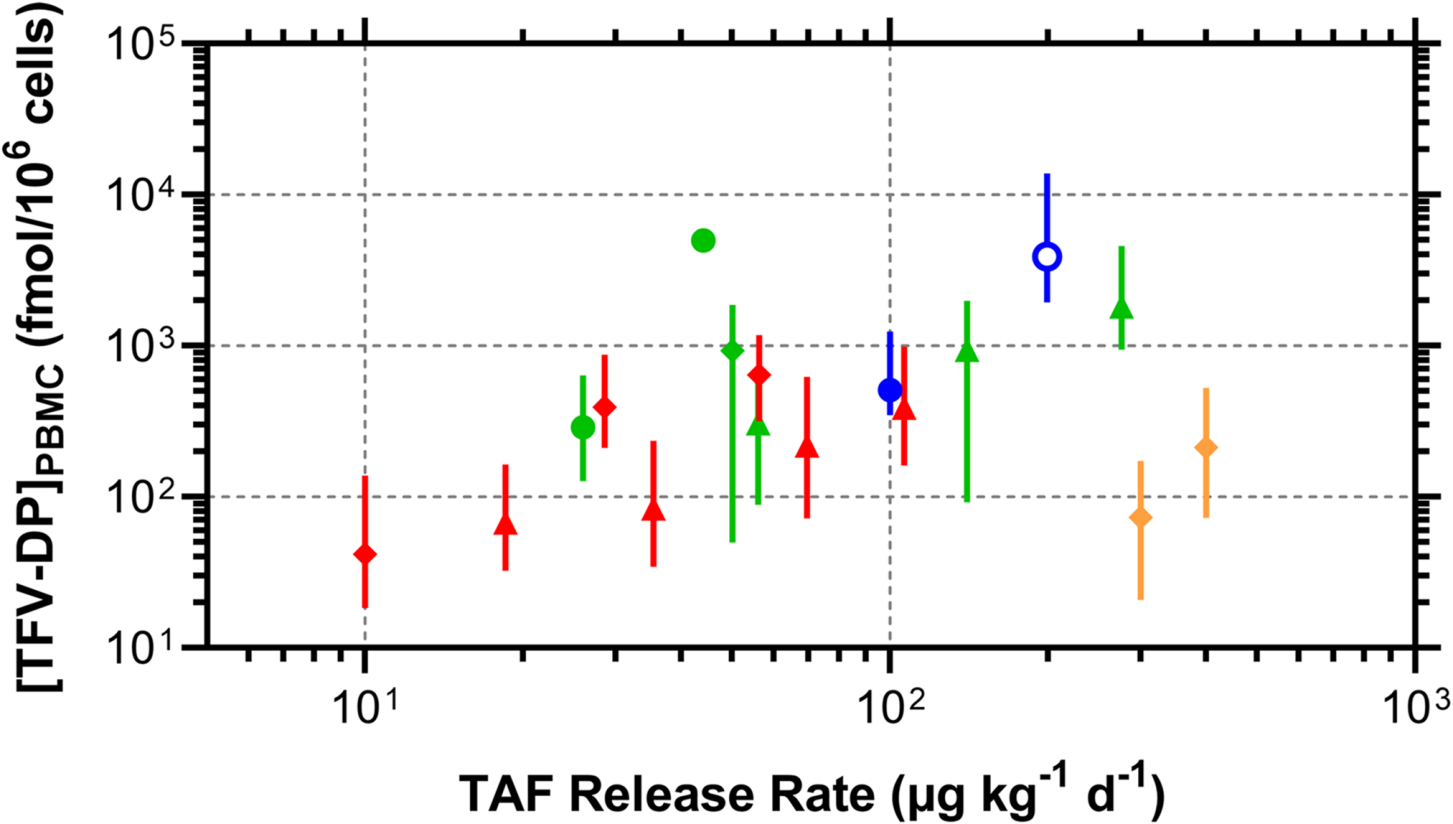
Relationship Between TAF *in Vivo* Release Rates (Body Weight Adjusted) and Equilibrium PBMC TFV-DP Concentrations (Medians, IQR) Differs Across Animal Species and Implant Technologies. Preclinical models are defined by symbol type: circles, beagle dogs; triangles, New Zealand white rabbits; diamonds, rhesus macaques. Implant technologies are defined by symbol color: blue, silicone reservoir implant with orifices [10] and current study; red, polyurethane reservoir implant [17]; green, poly(ε-caprolactone) reservoir implant [18, 24]; orange, transcutaneously refillable nanofluidic capsule implant [36, 40].

Given the focus of the current report and the wealth of multispecies PBMC TFV-DP equilibrium concentrations corresponding to a wide range of TAF implant technologies and animal models, we synthesized relevant preclinical data into one cohesive dataset (Fig. 9).

While there are general dose–response associations within a given species (on a log–log plot), these do not translate across species or studies. There could be practical reasons for this observation, such as different methods for collecting, counting, and processing PBMCs at the study sites, but most of the samples (PBMC lysates) were analyzed by the same laboratory at the Johns Hopkins University School of Medicine. There clearly are numerous complexities arising from the implant type, the form of the API (free-base or hemifumarate salt), and excipients that could impact the complex TAF pharmacology.

### Histological Description of the Subcutaneous Space

An understanding of the subcutaneous milieu is useful in the preclinical development of subdermal implants, and when comparing animal models. Histologically, mammalian skin is similar across most species and is comprised of three layers: the epidermis, the dermis, and the hypodermis [41–43]. The unique morphologies and functions of these three layers are manifested as differences in skin thickness, elasticity, hair density, and distribution of glands [44]. The epidermis is the outermost layer of the skin, functioning as an impermeable protective barrier to protect against the environment while also preventing water loss from the body, and has been described in detail by Kirschner *et al.* [45].

Below the epidermis is the dermis, a layer of mixed loose and dense layers of collagens and elastic fibers forming a network binding the skin to the underlying tissue, providing strength and elasticity to the organ [41–43, 46–49]. The connective tissue of the dermis varies between species, with some animals having a sparse layer of connective tissue, making the skin a relatively tight layer. In other animals, such as sheep and carnivores, the dermal connective tissue is loosely structured and plentiful allowing the skin to be mobile and flexible. The dermis is the site of hair follicles, glands (*e.g.*, sebaceous and sweat), blood capillaries, and sensory organs [42].

The hypodermis is the deepest layer of the skin and consists of irregular and haphazardly dispersed bundles of collagen and elastin fibers, forming a loose network binding the outer layers of the skin to the underlying tissues [42, 43]. The hypodermis also is well vasculated with larger blood capillaries than found in the dermis. The hypodermis can vary considerably between species due to the amount of collagen and fat cells (adipocytes and lipocytes) being present. Collagen concentrations are linked to the location of the skin and related to functionality.

Subdermal implants are placed through the epidermis to a site between the base of the dermal layer and the surface of the hypodermis. In a recent publication, we presented an ultrasound image from sheep where the implant can be seen under the dermis of the skin and above the hypodermis [13]. Implants in the subdermal layer are bathed in tissue fluid, originating from the cutaneous lymphatic system, rich in antigen presenting cells such as macrophages and dendritic cells [50, 51]. The histological similarities of skin from different mammals suggest that any differences between mammals in how they react to a drug and/or its mode of delivery may be due to factors at the cellular and sub-cellular level present in the lymphatic fluids surrounding the implanted device, and not only a result of structural differences. It is possible that subcellular differences between mammals influence the detectable differences in pH [52] and skin microbiome [53] on the outer surface of the skin. Such differences may provide more effective ways of screening for a model system than comparing histology alone.

## Conclusion

An innovative TAF subdermal implant system was evaluated *in vitro* and preclinically in three models: C57BL/6 J mice, merino sheep, and beagle dogs. Implant drug release was linear *in vitro* and *in vivo* and could be tuned over a wide range of release rates. Based on the data presented here, we recommend using mice for early-stage prototype implant testing and sheep as a large animal model for evaluation of lead candidates. Macaques serve as a complementary model that also can be used to obtain an efficacy endpoint. We found that dogs should be avoided as a large animal model due to extraneous *in vivo* release and PK results. However, these results could be device- or drug-specific and cannot be generalized. Our report provides fundamental, translatable insights into multispecies TAF delivery via long-acting implants.

## Data Availability

All other data supporting the findings of this manuscript are available from the corresponding author (MMB) upon reasonable request.
